# Supplementary material for: Joint modelling of systolic and diastolic blood pressure and its associated factors among women in Ghana: Multivariate response multilevel modelling methods
Source: PLOS Glob Public Health. 2023 Apr 26;3(4):e0001613. doi: 10.1371/journal.pgph.0001613 (PMC10132648; doi:10.1371/journal.pgph.0001613)
Supplement: Attachment — Submitted filename: Rebuttal Letter R1.pdf [file pgph.0001613.s001.pdf]

Joint modelling of systolic and diastolic blood pressure and its associated factors among women in Ghana: Multivariate response multilevel modelling methods

Manuscript ID: PGPH-D-22-01851

POINT-BY-POINT RESPONSES TO REVIEWS

We wish to express our heartfelt thanks and appreciation to the Editors and Reviewers for taking the time out of their tight schedules to review our manuscript and we found their comments very useful.

We have taken all the necessary steps to respond to your comments and thus, resubmit the revised manuscript for consideration and subsequent publication in your peer-reviewed journal. Our responses to your comments are presented below.

Editors' comments:

Journal Requirements:

1. Please review your reference list to ensure that it is complete and correct. If you have cited papers that have been retracted, please include the rationale for doing so in the manuscript text, or remove these references and replace them with relevant current references. Any changes to the reference list should be mentioned in the rebuttal letter that accompanies your revised manuscript. If you need to cite a retracted article, indicate the article's retracted status in the References list and also include a citation and full reference for the retraction notice.

Response: Thank you. We have done this, and also corrected those that are incomplete. See reference number 3, 6 and 22 in the reference list. In addition, we removed a duplicated reference number 28 which is the same as reference number 22 and corrected the remaining reference numbers starting from 28-32. See lines 426, 437 at page 23, lines 450, 452, 454, and 459 at page 24.

2. Please provide separate figure files in .tif or .eps format only and remove any figures embedded in your manuscript file. Please also ensure that all files are under our size limit of 10MB.

For more information about figure files please see our guidelines:

https://journals.plos.org/globalpublichealth/s/figures
https://journals.plos.org/globalpublichealth/s/figures#loc-file-requirement

Response: Thank you. We produced the figures as .tif file and per the PACE digital diagnostic tool as requested by the journal. We also revised the manuscript to remove the figures from the manuscript text. See pages 18-21.

3. Figs 1-4: please (a) provide a direct link to the base layer of the map (i.e., the country or region border shape) and ensure this is also included in the figure legend; and (b) provide a link to the terms of use / license information for the base layer image or shapefile. We cannot publish proprietary or copyrighted maps (e.g. Google Maps, Mapquest) and the terms of use for your map base layer must be compatible with our CC-BY 4.0 license.

Response: Thank you. First of all, we wish to state that all the maps were solely produced by the authors. The URL(s) or links to the base layer (shapefiles) of figures 1-4 and the terms of use/license information are available at https://gadm.org/download_country40.html and https://gadm.org/license.html. See lines 249-250 at page 10. The shapefiles are freely and openly available for download and use. Open access publication under CC-BY is allowed in PLoS as stated at under license at https://gadm.org/license.html.

Note: if you created the map in a software program like R or ArcGIS, please locate and indicate the source of the basemap shapefile onto which data has been plotted.

Response: Thank you. All the maps were solely produced by the authors of this manuscript using *tmap* package in *R* version 4.2.1. We earlier stated this in the manuscript. See line 251-252 at page 10. We earlier plotted the data for Figures 1-4 in *R* and selected the 'Esri.WorldGrayCanvas' basemap/tile. However, we have decided to remove the basemap/tiles from the maps. Thus, we produced new maps without the basemaps/tiles and replaced the old figures with the new figures. See Figures 1-4.

If your map was obtained from a copyrighted source please amend the figure so that the base map used is from an openly available source. Alternatively, please provide explicit written permission from the copyright holder granting you the right to publish the material under our CC-BY 4.0 license.

Response: Thank you. We did not obtain our maps from a copyrighted source. The authors solely produced these maps for this manuscript.

Please note that the following CC BY licenses are compatible with PLOS license: CC BY 4.0, CC BY 2.0 and CC BY 3.0, meanwhile such licenses as CC BY-ND 3.0 and others are not compatible due to additional restrictions.

Response: Thank you. The license for the shapefile is compatible with PLoS license. See https://gadm.org/license.html. For the plotted maps, no license is required because the maps were solely produced by the authors for this manuscript, and we did not use any basemaps/tiles in the current figures.

If you are unsure whether you can use a map or not, please do reach out and we will be able to help you. The following websites are good examples of where you can source open access or public domain maps:
* U.S. Geological Survey (USGS) - All maps are in the public domain. (http://www.usgs.gov)
* PlaniGlobe - All maps are published under a Creative Commons license so please cite "PlaniGlobe, http://www.planiglobe.com, CC BY 2.0" in the image credit after the caption. (http://www.planiglobe.com/?lang=enl)
* Natural Earth - All maps are public domain. (http://www.naturalearthdata.com/about/terms-of-use/)

Response: Thank you. This does not apply to our manuscript based on the earlier response provided.

Review Comments to the Author:

Reviewer #1: there are a couple of areas that need editorial work. some paragraphs are too long, some sentences are a mouthful. just basic edits. This is especially so in the discussion and conclusions sections of the manuscript

Response: Thank you. We have addressed these issues across the manuscript. For example, see lines 402, 419, 420 at page 22, 477, 478, 491,492 at page 25.

Reviewer #2: Title of manuscript: Joint modelling of systolic and diastolic blood pressure and its associated factors among women in Ghana: Multivariate response multilevel modelling methods
The various factors affecting blood pressure in women of childbearing age is an important topic in public health as hypertension can lead to other cardiovascular diseases and mortality as well. Understanding the causative factors that lead to high blood pressure can help prevent many people from being hypertensive. The study was done on 4744 women residing in 3722 households using data from the 2014 Ghana Demographic and Health Survey. The authors recommend interventions to be targeted at high-risk groups including older generation and those who are obese.
Though the topic fits into the scope of the journal, the study has few weaknesses that needs attending to. The abstract section should be subtitled. There are some spelling and grammatical errors which needs correction. Some references are incomplete, such as reference number 3. Overall, the manuscript is well written.

Response: Thank you. The journal's abstract formatting is what we have used, where no subtitle of the abstract is required. Thus, the journal employs continuous paragraph and no breaks for the abstract. As a result, we did not make any changes to the abstract. We have also addressed the reference issues (see reference number 3, 6 and 22 in the reference list) and have done proofreading across the manuscript. See specific responses below:

Introduction

Page 1. Introduction. First paragraph
Hypertension is a significant risk factor for cardiovascular diseases, including stroke, myocardial infraction, heart failure, and atherosclerosis, which account for most of the non-communicable disease deaths globally. Change 'infraction' to 'infarction'.

Response: Thank you. We revised the manuscript to address this. See line 78 at page 3.

Page 2. Introduction. First paragraph
Understanding the influence of these demographic and social-ecological factors on hypertension is critical to addressing them, reducing prevalence, and improving treatment outcomes. Replace 'social-ecological' with 'socio-ecological'.

Response: Thank you. We revised the manuscript to address this. See line 121 at page 5.

Page 2. Introduction. Last paragraph
The multivariate response multilevel modelling is critical in this study because we are interested in modelling both SBP and SBP outcomes measured on same woman nested

within a household and we need to draw conclusions about the degree to which the residual correlations depend on these women and their households. What about DBP?

Response: Thank you. We revised the manuscript to address this. See line 136 at page 5.

Materials and methods
Statistical Analysis: Second paragraph
Insert ‘high’ blood pressure. The sentence should read as ‘Clinically, measurement of high blood pressure/hypertension is based on the combination of systolic blood pressure and diastolic blood pressure.’

Response: Thank you. We revised the manuscript to address this. See line 189 at page 8.

Ethics Approval and Consent to Participate
An informed consent statement is read to the respondent, who may accept or decline to participate. Write this sentence in past tense.

Response: Thank you. We revised the manuscript to address this. See line 260 at page 11.
