# Supplementary material for: Joint modelling of systolic and diastolic blood pressure and its associated factors among women in Ghana: Multivariate response multilevel modelling methods
Source: PLOS Glob Public Health. 2023 Apr 26;3(4):e0001613. doi: 10.1371/journal.pgph.0001613 (PMC10132648; doi:10.1371/journal.pgph.0001613)
Supplement: Attachment — Submitted filename: Rebuttal Letter R2.pdf [file pgph.0001613.s002.pdf]

Joint modelling of systolic and diastolic blood pressure and its associated factors among women in Ghana: Multivariate response multilevel modelling methods

Manuscript ID: PGPH-D-22-01851R1

POINT-BY-POINT RESPONSES TO REVIEWS

We wish to express our heartfelt thanks and appreciation to the Editors and Reviewers for taking the time out of their tight schedules to review our manuscript and we found their comments very useful.

We have taken all the necessary steps to respond to your comments and thus, resubmit the revised manuscript for consideration and subsequent publication in your peer-reviewed journal. Our responses to your comments are presented below.

Editors' comments:

Journal Requirements:

1. Please review your reference list to ensure that it is complete and correct. If you have cited papers that have been retracted, please include the rationale for doing so in the manuscript text, or remove these references and replace them with relevant current references. Any changes to the reference list should be mentioned in the rebuttal letter that accompanies your revised manuscript. If you need to cite a retracted article, indicate the article's retracted status in the References list and also include a citation and full reference for the retraction notice.

Response: Thank you. We have done this, and all the references are complete. We did not find any of our references retracted.

Review Comments to the Author:

Reviewer #1: General remarks:
A number of issues raised earlier have been addressed and so left out in these new comments. Generally, the manuscripts looks much better now. A few areas here below may need address.

Response: Thank you once again for critical assessment of our manuscript. We have addressed the comments point by point as below:

Abstract
1. I suggest dropping the less prevalent significant predictors of BP such as toilet type and cooking fuel from the abstract. They are curious and the abstract doesn't provide for synthesis of how they affect BP. Maybe you only maintain the more prevalent predictors in abstract?

Response: Thank you. We revised the manuscript to reflect this. See lines 52-53 and line 56 at page 2.

Introduction

1. Line 76. Authors say that when blood pressure is too high it exerts force on arterial walls thus leading to a critical reduction in blood flow. This is inaccurate. The worry in high blood pressure and hypertension is primarily destruction of blood vessels. If the author chooses to go that direction, they must discuss the physiologic mechanisms entailed.

Response: Thank you very much for the feedback. Our goal was to simply define SBP and DBP measurements. We revised the manuscript to address this for clarity which was supported with a reference from WHO. See lines 80-82 at page 3.

Materials and methods

1. While there is probably no way out for the authors, data used is 8 years old and it is likely the described predictors have changed since. This is weakened further by the mention that such DHS data is available every 5 years. Was there any DHS report after 2014?

Response: Thank you. This is the most recent DHS data available for Ghana at the time of the study. The next round of the DHS survey for Ghana is currently ongoing and likely to be completed and released by next year. There is therefore no way out for the authors as indicated by the reviewer.

Results.

1. Line 272-275. The demographic information like mean age, numbers per age bracket, numbers with different levels of education, etc is lacking. This would go a long way in making readers appreciate the direction of the discussion being built.

Response: Thank you. We revised the manuscript to address this. See lines 276-280 and Table 1 at page 12.

2. Line 274. Paragraph one has a statement “However, a reduced figure of 6.9% of the women said they were ever told to have had hypertension” which is unclear.

Response: Thank you. We revised the manuscript to address this. See line 275 at page 11.

3. Figure 3 needs some editorials (“observed” instead of “observe”)

Response: Thank you. We revised the manuscript to address this. See line 369 at page 19.

Discussion

1. Line 404. The statement “The hypertension prevalence based on SBP, DBP, both SBP and DBP, and reported were 7.1%, 10.2%, 12.3%, and 6.9% respectively, with substantial regional differences in the hypertension prevalence for all the four measures” needs revision.

Response: Thank you. We revised the manuscript to address this. See lines 385-390 at page 19.

2. The discussion section has a lot of repeated results. Kindly revise this.

Response: Thank you. We revised the manuscript to address this. See lines 398-399 at page 20.

3. I was keen to see what discussion would come from the significant findings on toilet type and cooking fuel and how this fits in the larger scope on the topic but this is missing. For the other significant aspects as well, I wish likely mechanisms would have been discussed to make this work fit better in addressing a global public health problem from an understanding of the associated predictors.

Response: Thank you. We revised the manuscript to address this. See lines 443-449 at page 22.

Conclusion
1. This section is too long. It also has unnecessary details that would probably fit elsewhere (within discussion).

Response: Thank you. We revised the manuscript to address this. See lines 488-502 at page 24.

2. Some conclusions (like need to do screening and etc) are not drawn from the scope of the current study

Response: Thank you. We removed this from the manuscript. See lines 499-502 at page 24
